# Genomic and physiological variability within Group II (non-proteolytic) *Clostridium botulinum*

**DOI:** 10.1186/1471-2164-14-333

**Published:** 2013-05-16

**Authors:** Sandra C Stringer, Andrew T Carter, Martin D Webb, Ewelina Wachnicka, Lisa C Crossman, Mohammed Sebaihia, Michael W Peck

**Affiliations:** 1 Institute of Food Research (IFR), Norwich Research Park, Colney, Norwich NR4 7UA, UK; 2 The Genome Analysis Centre (TGAC), Norwich Research Park, Norwich NR4 7UH, UK; 3 Welcome Trust Sanger Institute, Hinxton, Cambridge CB10 1BA, UK; 4 Present address: Departement de Biologie, Faculté des Sciences,Université Hassiba Ben Bouali, Chlef, Algeria

## Abstract

**Background:**

*Clostridium botulinum* is a group of four physiologically and phylogenetically distinct bacteria that produce botulinum neurotoxin. While studies have characterised variability between strains of Group I (proteolytic) *C. botulinum*, the genetic and physiological variability and relationships between strains within Group II (non-proteolytic) *C. botulinum* are not well understood. In this study the genome of Group II strain *C. botulinum* Eklund 17B (NRP) was sequenced and used to construct a whole genome DNA microarray. This was used in a comparative genomic indexing study to compare the relatedness of 43 strains of Group II *C. botulinum* (14 type B, 24 type E and 5 type F). These results were compared with characteristics determined from physiological tests.

**Results:**

Whole genome indexing showed that strains of Group II *C. botulinum* isolated from a wide variety of environments over more than 75 years clustered together indicating the genetic background of Group II *C. botulinum* is stable. Further analysis showed that strains forming type B or type F toxin are closely related with only toxin cluster genes targets being unique to either type. Strains producing type E toxin formed a separate subset. Carbohydrate fermentation tests supported the observation that type B and F strains form a separate subset to type E strains. All the type F strains and most of type B strains produced acid from amylopectin, amylose and glycogen whereas type E strains did not. However, these two subsets did not differ strongly in minimum growth temperature or maximum NaCl concentration for growth. No relationship was found between tellurite resistance and toxin type despite all the tested type B and type F strains carrying *tehB*, while the sequence was absent or diverged in all type E strains.

**Conclusions:**

Although Group II *C. botulinum* form a tight genetic group, genomic and physiological analysis indicates there are two distinct subsets within this group. All type B strains and type F strains are in one subset and all type E strains in the other.

## Background

*Clostridium botulinum* is an important pathogen as it forms the highly potent botulinum neurotoxin that is responsible for botulism. It is a physiologically and genetically heterogeneous species within which four metabolically and genetically distinct groups are recognised [1]. Cases of human botulism are usually associated with Group I or Group II strains. Group I (proteolytic) *C. botulinum* is a mesophilic bacterium with a minimum growth temperature of 10-12°C, an optimum of 37°C and maximum growth temperature of around 48°C. It is highly proteolytic, forms very heat resistant spores and strains form one, or occasionally two, neurotoxins of types A, B or F. Group II (non-proteolytic, saccharolytic) *C. botulinum* is a psychrotrophic bacterium with a minimum growth temperature of 2.5-3.0°C and an optimum of 30°C. It ferments a number of sugars, forms spores with moderate heat resistance, and strains form a single neurotoxin of type B, E, or F [2]. Group II *C. botulinum* is often associated with outbreaks of foodborne botulism involving fish and meat [3] and is a concern in the continued safe production of chilled ready meals [4].

The variability and relationships between strains, and particularly between the different toxin serotypes, within Group II *C. botulinum* are not well understood. Several authors have used a range of methods to type *C. botulinum* including 16s rRNA [5,6], AFLP [6,7], pulsed-field gel electrophoresis (PFGE) [8,9], randomly amplified polymorphic DNA (RAPD) [9] and ribotyping [10,11]. However, these studies have either concentrated on the genetic diversity of the entire *C. botulinum* species or diagnostic subtyping of closely related strains. More recently Macdonald et al. [12] used MLST, AFLP, VNTR and sequencing of the neurotoxin gene to examine the relationships between 41 type E strains. None of these studies have included more than a total of six strains of either type B or type F.

Hyyatia et al. [13] tested 21 strains of Group II *C. botulinum* including 5 type B, 3 type F and 13 type E in an RAPD and rep-PCR analysis, and Kirkwood et al. [14] included 4 type B, 6 type F and 24 types E strains in their focal plane array Fourier transform infrared (FPA-FTIR) and PFGE analysis, but both these papers were aimed at diagnostic subtyping rather than extending understanding of strain variability.

The aim of the present study was to improve understanding of the diversity and clustering of strains of Group II *C. botulinum* by examining both the genomics and the physiology of strains*.* We analysed a newly sequenced genome and compared it with other genomes of Group II *C. botulinum* that are publically available. We have also constructed a microarray suitable for comparative genomics based on the sequence of Group II *C. botulinum* Eklund 17B (NRP), and used this to carry out the first reported comparative genomic indexing study of Group II *C. botulinum* which included 14 type B, 24 type E and 5 type F strains. Physiological analyses have included measurement of the maximum NaCl concentration and minimum temperature permitting growth as well as carbohydrate utilisation and tellurite resistance.

## Results

### Sequence data

The genome features of Group II *C. botulinum* Eklund 17B (NRP) are shown in Table 1. The genome of strain Eklund 17B (NRP) consists of a chromosome of 3,781,509 bp and a plasmid of 47,689 bp. The chromosome carries 3,462 coding sequences and has a GC content of 27%. The plasmid carries 55 coding sequences and has a GC content of 25%.

**Table 1:** **Characteristics of whole genome sequences of Group II *C. botulinum *strains**

**Strain**	**Eklund 17B (NRP)**	**Eklund 17B (JGI)**	**Alaska E43**	**Beluga**
Chromosome				
Length (bp)	3 781 509	3 800 327	3 659 644	3 999 203
Number of CDSs	3 462	3 425	3 257	3 684
%GC content	27.44	27.51	27.36	27.44
Coding density genes/kb	0.915	0.901	0.889	0.921
Average gene length (bp)	903	906	926	887
Coding percentage (%)	82.7	81.7	82.4	81.7
Plasmid pCLL				
Length (bp)	47 689	47 642	Absent	Absent
%GC content	24.95	24.95		
Number of CDSs	55	50		

### Comparison with a previously released Eklund 17B genome sequence

During the sequencing and assembly of the Eklund 17B (NRP) genome a complete finished sequence of another isolate of strain Eklund 17B was made available by the Joint Genome Institute, USA (JGI), accession number A28857. Strain Eklund 17B was originally isolated in 1965 but the two sequenced isolates had been in separate laboratories for at least 25 years. Genomic comparisons of the two sequences reveal a small number of single nucleotide polymorphisms (SNPs), 45 in total, in addition to some potential assembly differences (detailed in Additional file 1: Table S1). The potential assembly differences largely relate to the ribosomal RNA operons. These repetitive and virtually identical regions are notoriously difficult to assemble and therefore the observed changes may represent differences in the assembly rather than differences in the target genomic DNA sequence. The SNPs may have resulted from the use of different platforms, methodologies and software, however, it cannot be ruled out that genetic changes have occurred during the period of separation.

#### Genome comparisons with other Group II C. botulinum strains

Only two additional whole genome sequences of Group II *C. botulinum* strains are currently available in the sequence databases. These are type E3 toxin producing strain Alaska E43 and type E1 toxin producing strain Beluga, accession numbers NC_010723 and ACSC01000000 respectively. The sequences for strain Alaska E43 is finished to completion, while the sequence for strain Beluga is a draft assembly comprising six contigs. The available Group II *C. botulinum* genomes were downloaded and used in downstream analysis of the Eklund 17B (NRP) genome.

**Figure 1:**
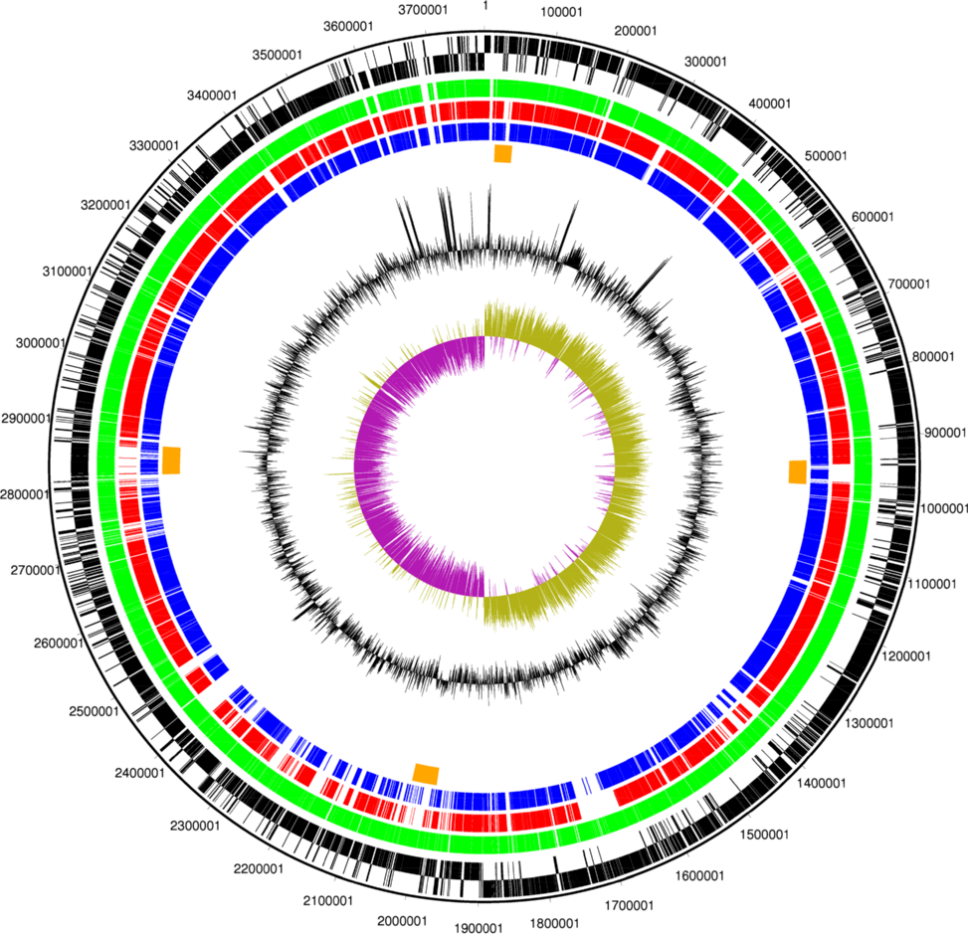
**Circular figure showing relatedness of the chromosome of sequenced Group II *C. botulinum *strains.** Concentric rings represent the genomes of Eklund 17B (NRP) and three other sequenced strains of Group II *C. botulinum*. From outermost to innermost ring: 1. Black: DNA coordinates for Eklund 17B (NRP) DNA sequence 2. Black: Forward and reverse frames coding sequences for Eklund 17B (NRP). 3. Green: Orthologous coding sequences for Eklund 17B (JGI) (forward only). 4. Red: Orthologous coding sequences shared with Alaska E43 (forward only). 5. Blue: Orthologous coding sequences shared with Beluga (forward only). 6. Orange: Predicted intact bacteriophage sequences in Eklund 17B (NRP). 7. Black: GC content plot as a percentage for Eklund 17B (NRP). 8. Purple/green: GC skew plot for Eklund 17B (NRP). The figure indicates the rRNA operons as regions containing no coding sequences and also represented by a clear spike in the GC content plot present near the replication origin and termination regions. The GC skew plot shows a positive skew on the leading strand.

CDSs shared between strains Eklund 17B (NRP), Alaska E43 and Beluga were determined by reciprocal best match analysis. The general features of all three genomes are summarised in Table 1 and a circular representation of the genome showing orthologous genes shared between the strains is shown in Figure 1. Figure 1 shows specific regions of variability amongst the strains, which may represent insertions into the backbone DNA of the organism.

Phage DNA was modelled by the Phast program [15]. Four intact prophages were predicted in the Eklund 17B (NRP) genome. These were designated as sharing the highest similarity with Geobacillus virus E2, Clostridium phage Φ3626, Clostridium phage ΦCD119 and Clostridium phage ΦCD27. An additional potential prophage was described as questionable but sharing the highest similarity with Clostridium phage ΦCD6356. In comparison, Alaska E43 was predicted to possess only one intact phage region; this was located at a similar genetic position to the phage predicted as questionable in Eklund 17B (NRP). By contrast the Beluga genome was predicted to contain three large intact prophages, one of which shares the highest similarity with Clostridium phage ΦCD1119 and which is present in a similar genetic position to the equivalent prophage in Eklund 17B (NRP). The other two predicted Beluga prophages were present at different positions to those in Eklund 17B (NRP). At approximately 1,700,000 bp in the Eklund 17B (NRP) genome, there are a number of CDSs for phage proteins that are shared with Beluga but not with Alaska E43. These do not constitute an intact prophage in either Eklund 17B (NRP) or Beluga.

**Figure 2:**
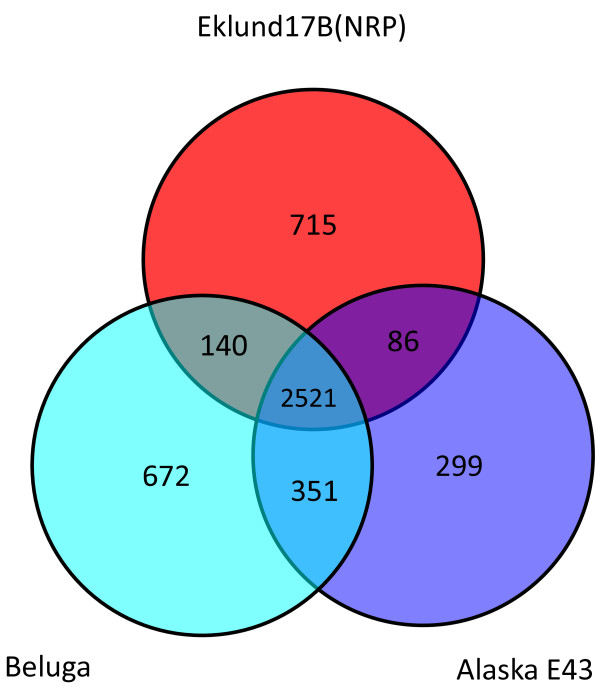
**Venn diagram showing the relatedness of Group II *C. botulinum *strains.** Orthologous CDSs were calculated between the three available Group II *C. botulinum* genome sequences by reciprocal FASTA best match analysis. CDSs shared between specific strains and CDSs unique to a particular strain are plotted on the Venn diagram.

Regions that were unique to the Eklund 17B (NRP) genome but were not associated with phage were also detected. Many of these regions carry CDSs for enzymes involved in metabolic pathways, regulators and transporters. Other regions specify cell envelope components. Specific unique CDSs include cold shock and heat shock proteins. A CDS for a predicted protein with similarity to an endolysin from *C. beijerinckii,* a septicolysin similar to that from *C. septicum* and a CDS sharing significant similarity with an internalin from *Listeria ivanovii* is also present. A Venn diagram was formulated to represent the number of CDS shared between the three sequenced Group II *C. botulinum* strains and the numbers of unique CDS (Figure 2). A total of 2521 CDS were common to all three strains out of a total of between 3257 and 3684 CDS (Figure 2 and Table 1).

### Features of Plasmid pCLL

**Figure 3:**
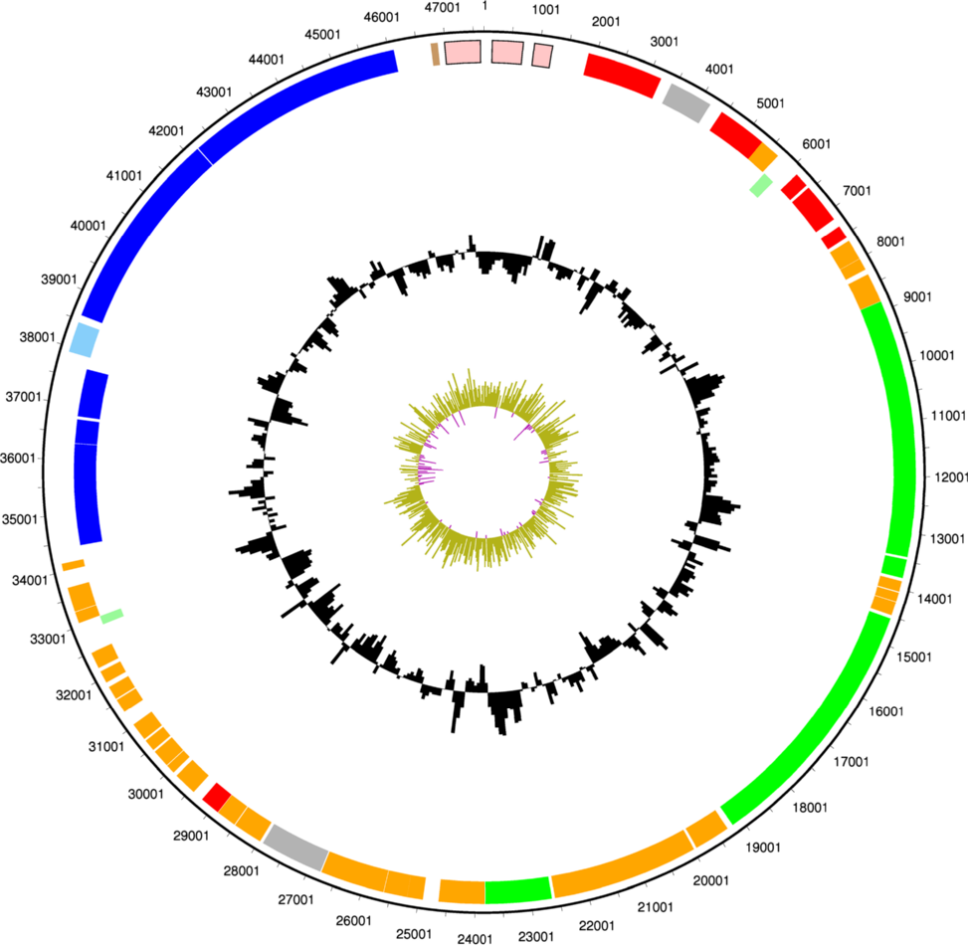
**Circular figure showing features of Group II *C. botulinum *Eklund 17B (NRP) plasmid pCLL.** Concentric rings show the following from outermost to innermost. 1–2. Plasmid features. Orange indicates conserved hypothetical CDSs, red indicates CDSs involved in plasmid replication and maintenance, royal blue indicates CDSs in the toxin gene cluster, light blue indicates regulatory protein, pink indicates mobile element features, green indicates predicted transmembrane proteins, brown indicates a pseudogene and light green indicates hypothetical proteins with no significant database hits. 3. GC content plot as a percentage 4. GC skew plot.

**Figure 4:**
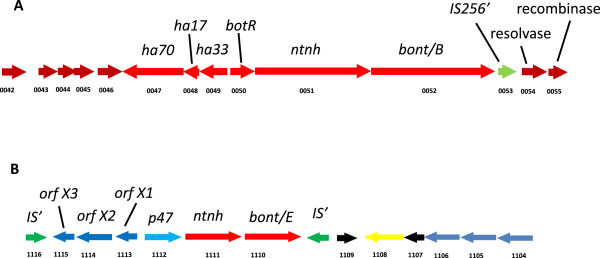
**Organisation of the botulinum neurotoxin clusters of Group II *C. botulinum *Eklund 17B (NRP) and Alaska E43.***C. botulinum* neurotoxin genes (bont) are located with other associated proteins in one of two types of cluster arrangement. In Group II *C. botulinum* all published type B neurotoxin genes are in a *ha* cluster and all type E and type F neurotoxin genes are in an orfX cluster. Eklund 17B and Alaska E43 show typical cluster organisation. **A**. The neurotoxin gene of Group II *C. botulinum* type B strain Eklund 17B (NRP) is in a *ha* cluster located on a plasmid, CDS numbers CB17B_P047 to CB17B_P052. **B**. The published sequence of Group II *C. botulinum* type E strain Alaska E43 (accession number NC_010723) shows that the neurotoxin gene is located on the chromosome in an *orfX* cluster, CDS CLH_1110 to CLH_1115.

The genome of Eklund 17B (NRP) includes one plasmid which carries the genes of the toxin cluster. The plasmid features are described Figure 3. There are two conserved botulinum neurotoxin cluster types, the *ha* cluster and the *orfX* cluster [1]. The toxin gene clusters from Group II *C.botulinum* type B strain Eklund 17B (NRP) and type E strain Alaska E43 (accession number NC_010723) are shown in Figure 4 illustrating the typical organisation of the *ha* cluster and *orfX* cluster types respectively. The toxin gene of *C.botulinum* Eklund 17B (NRP) is in a *ha* cluster that sits adjacent to a resolvase-recombinase that may be significant in the lateral gene transfer of this cluster to alternative plasmid and chromosomal locations. Specific features of the plasmid include plasmid replication and maintenance CDSs and several conserved hypothetical CDSs of similar length. BLAST analysis shows that, for the most part, these CDSs only show significant sequence similarity to the equivalent genes from Eklund 17B (JGI). These CDSs do not show any conserved Pfam domain features or predicted transmembrane domains. Repeat analysis using the program REPuter [16] does not indicate that these genetic regions are highly repetitive, although repeats are detectable on the plasmid.

### Comparative genomic indexing of strains using Microarray

**Figure 5:**
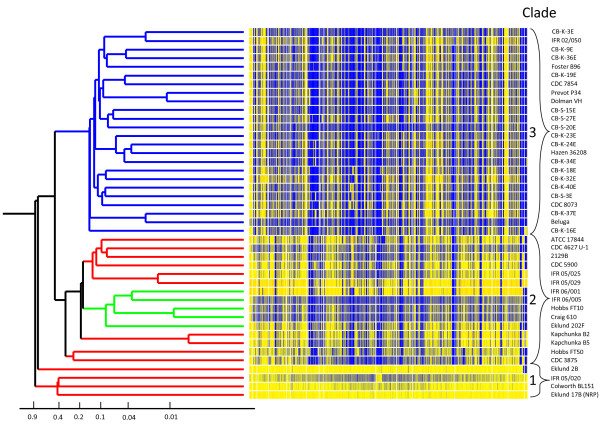
**Genomic indexing of 43 strains of Group II *C. botulinum.*** An average linkage hierarchical clustering dendrogram of the *C. botulinum* strains was created in GeneSpring version 7.0 from microarray data using the Pearson coefficient correlation. Each row of the heatmap represents one strain and consists of a series of vertical bars which represent the CDS content of *C. botulinum* Eklund 17B (NRP). From left to right these bars are the chromosome from CB17B_0001 to CB17B_3470 then the plasmid from CB17B_P01 to CB17B_P54. The colour of each bar indicates the ratio of the signal from the test strain over that of the index strain. Where the signal from DNA binding is equal for both the test and control strains the bar is coloured yellow and where there is lower binding by the test strain the bar is blue. Where there is no probe the bar is white. The hierarchical clustering dendrogram has been coloured according to neurotoxin type: red =type B, blue =type E and green = type F. Strains were divided into three clusters using a distance of 0.5. Strain Eklund 2B appears slightly separate from the other Cluster 1 strains but this was a result of the culture used on the array lacking the 54 plasmid CDSs. Using only the chromosomal probes, Eklund 2B clustered with the other three strains in Clade 1.

The DNA microarray used for competitive genomic indexing in this study comprised 4160 probes. Each was sixty nucleotides long and tested by BLAST analysis to bind to unique sites in the Group II *C. botulinum* Eklund 17B (NRP) chromosome and plasmid sequences. These probes represented 3384 chromosomal CDSs and 50 plasmid CDSs which is 97.4% of the 3524 total CDSs that were predicted from the sequence data available at that time. This microarray was used to index the CDS content of 43 strains of Group II *C. botulinum* in relation to the control Eklund 17B (NRP) strain. The hybridization intensity ratio of genomic DNA from the test strain hybridized against Eklund 17B (NRP) DNA for each CDS in chromosome order is shown in Figure 5. The number of CDSs where the test probe signal was within 8-fold of the control signal is shown in Table 2.

**Table 2:** **Percentage of CDSs from chromosome of Eklund 17B (NRP) scored positive by microarray analysis of strains of Group II *C. botulinum***

**Strain**	**Toxin subtype**	**Clade**	**Total number of CDSs present**	**% of control CDSs present**
Eklund 17B (NRP)	B4	1	3384	100
Eklund 2B	B4	1	3384	100
IFR 05/020	B4	1	3383	100
Colworth BL151	B4	1	3383	100
IFR 05/029	B4	2	2959	87
IFR 06/001	F6	2	2917	86
IFR 05/025	B4	2	2900	86
Eklund 202F	F6	2	2863	85
2129B	B4	2	2848	84
CDC 5900	B4	2	2846	84
ATCC 17844	B4	2	2845	84
Hobbs FT50	B4	2	2829	84
Kapchunka B2	B4	2	2806	83
CDC 4627 U-1	B4	2	2792	83
Kapchunka B5	B4	2	2777	82
Hobbs FT10	F6	2	2761	82
Craig 610	F6	2	2758	82
IFR 06/005	F6	2	2750	81
CDC 3875	B4	2	2716	80
CB-K-37E	E1	3	2428	72
CB-K-32E	E6	3	2424	72
CB-K-36E	E6	3	2404	71
CB-K-24E	E1	3	2403	71
IFR 02/050	E3	3	2403	71
CDC 8073	E2	3	2401	71
CB-K-23E	E1	3	2351	69
Foster B96	E3	3	2292	68
CB-K-16E	E1	3	2281	67
CB-S-3E	E1	3	2276	67
CB-K-40E	E3	3	2263	67
CB-S-27E	NT^E^	3	2249	66
CB-K-18E	E1	3	2242	66
Prevot P34	E3	3	2164	64
CB-K-34E	E1	3	2120	63
CB-S-20E	NT^E^	3	2095	62
CB-S-15E	NT^E^	3	2072	61
Dolman VH	E3	3	2064	61
Hazen 36208	E1	3	2058	61
CB-K-9E	E6	3	2024	60
CB-K-3E	E3	3	2023	60
CDC 7854	E1	3	2012	59
Beluga	E1	3	1990	59
CB-K-19E	E1	3	1946	58

Sixty-mer probes were used to represent 3384 chromosomal sequences of Eklund 17B (NRP) on a microarray. Data show the number of these CDSs found in each test strain by microarray analysis. CDSs were considered to be present if the test probe signal was within 8-fold of the control probe signal and significantly above the background. NT^E^= no toxin gene detected but strain originally isolated containing type E neurotoxin gene.

Hierarchical clustering was performed to assess the genomic relatedness of the strains (Figure 5). The branch lengths represent the linkage distance between strains calculated using a Pearson correlation pairwise comparison function. The clustering analysis divided Group II *C. botulinum* into three main clades using a distance marker of 0.5. Clade 1 included the control strain, Eklund 17B (NRP), and three other type B strains. Clade 2 contained the remainder of the type B strains and all the type F strains. All type E strains were together in clade 3.

The four strains in Clade 1 all scored positive for the same chromosomal CDSs as the test Eklund 17B (NRP) strain. Three strains also contained the plasmid CDS, including the toxin cluster, but these were not present in strain Eklund 2B. As Eklund 2B was initially able to produce toxin but did not contain the toxin genes sequence when tested on the microarray, it had presumably lost the plasmid during culture or preparation. This lack of the Eklund 17B (NRP) plasmid CDSs makes Eklund 2B appear slightly separated from the other Cluster 1 strains on the dendrogram (Figure 5). Using only chromosomal probes, Eklund 2B clustered with the other three strains in the Eklund 17B (NRP) clade.

In general, no relationship was observed between the clades and geographical origin, environment, food type or isolation date. However, many pairs of strains were very similar to each other when compared to the genome of Eklund 17B (NRP). In some cases these pairs of strains had been isolated in a short time from similar samples. For example, type B strains IFR 05/025 and IFR 05/029 were both isolated from egg pastas in 2005, type B strains Eklund 17B and Eklund 2B were both isolated from Pacific sediment in 1965, and type B strains Kapchunka B2 and Kapchunka B5 were both isolated from salted fish (kapchunka) in 1981. In other cases strains from geographically and chronologically diverse isolations also appear similar such as type F strains Craig 610 and Hobbs FT10, type E strains Beluga and CB-K-37E, and type E strains Dolman VH and Prevot P34 (Figure 5).

### *C. botulinum* neurotoxin gene subtyping and toxin cluster genes

The neurotoxin genes of *C. botulinum* are found in one of two conserved cluster types, the *ha* cluster and the *orfX* cluster (for examples see Figure 4) generally with a single neurotoxin gene per cluster. All the strains of Group II *C. botulinum* listed in Table 3 were tested on a second microarray designed using probes to published *ha* cluster genes or *orfX* cluster genes. Most of the available toxin cluster gene sequences available at that time were from Group I or Group III *C. botulinum* species. Unfortunately these probes were not particularly effective for binding the Group II *C. botulinum* strains used in this study and therefore could not be used to distinguishing between them. For example, although the type F toxin is associated with the *orfX* cluster, DNA from Group II *C. botulinum* type F strains did not show strong binding to any of the *orfX* gene probes which were generated from Group I toxin cluster sequences.

**Table 3:** **Details of Group II *Clostridium botulinum *strains analysed**

**Toxin subtype**	**Strain name**	**Isolation details**			**Linked to Outbreak**	Received from^*^
		**Source**	**Location**	**Date**		
B4	Eklund 17B	Pacific sediments	USA	1965	no	NCIMB
B4	Eklund 2B	Pacific sediments	USA	1965	no	UR
B4	Colworth BL151	Haddock	Norway	1960s?	no	UR
B4	IFR 05/020	Scallops	Canada	2005	no	IFR
B4	Hobbs FT50	Herring	UK	1960s?	no	UR
B4	CDC 3875	Human stool from botulism case	Iceland	1981	yes	CDC
B4	CDC 4627 U-1	Whey used to pickle/store blood sausage	Iceland	1983	yes	CDC
B4	Kapchunka B2	Dried salted whole whitefish [Kapchunka]	USA	1981	yes	NFPA
B4	Kapchunka B5	Dried salted whole whitefish [Kapchunka]	USA	1981	yes	NFPA
B4	2129B	Unknown	France	1950s?	?	NFPA
B4	CDC 5900	Human stool from botulism case	Italy	1986	yes	CDC
B4	ATCC 17844	unknown	unknown	unknown	?	UH
B4	IFR 05/025	Dried egg pasta (Fettucine)	UK	2005	no	IFR
B4	IFR 05/029	Dried egg pasta (Trucioli)	UK	2005	no	IFR
E1	Beluga	Fermented Beluga whale [Muktuk]	USA	1950	yes	UR
E3	Foster B96	Smoked fish	USA	1960s	?	UR
E1	Hazen 36208	Labrador smoked salmon	USA	1934	yes	NCIMB
E3	Prevot P34	Pond-reared freshwater perch	France	1951	no	UR
E3	Dolman VH	Pickled herring	Canada	1949	yes	CDC
E1	CDC 7854	Dried salted whole mullet [Faseikh]	Egypt	1991	yes	CDC
E2	CDC 8073	Human stool from botulism case	Alaska, USA	1991	yes	CDC
E3	IFR 02/050	unknown	UK	2002	No	IFR
E3	CB-K-3E	Rainbow trout surface	Finland	1995	no	UH
E6	CB-K-9E	Rainbow trout intestines	Finland	1995	no	UH
E1	CB-K-16E	Rainbow trout intestines	Finland	1995	no	UH
E1	CB-K-18E	Lake trout intestines	Finland	1995	no	UH
E1	CB-K-19E	Frozen whitefish roe	Finland	1996	no	UH
E1	CB-K-23E	Burbot intestines	Finland	1996	no	UH
E1	CB-K-24E	Burbot surface	Finland	1996	no	UH
E6	CB-K-32E	Vendace	Finland	1996	no	UH
E1	CB-K-34E	Frozen whitefish roe	Finland	1996	no	UH
E6	CB-K-36E	Vacuum-packed cold smoked rainbow trout	Finland	1996	no	UH
E1	CB-K-37E	Vacuum-packed hot smoked whitefish	Finland	1996	no	UH
E3	CB-K-40E	Vacuum-packed cold smoked rainbow trout	Finland	1996	no	UH
E1	CB-S-3E	Sediment mud	Finland	1995	no	UH
E^NT^	CB-S-15E	Fishfarm sediment	Finland	1997	no	UH
E^NT^	CB-S-20E	Fishfarm sediment	Finland	1997	no	UH
E^NT^	CB-S-27E	Fishfarm sediment	Finland	1997	no	UH
F6	Eklund 202F	Pacific sediments	USA	1965	no	UR
F6	Hobbs FT10	Atlantic Herring from Moray Firth	UK	1970s	no	UR
F6	Craig 610	Salmon from Columbia River	USA	1965	no	UR
F6	IFR 06/001	Scallops	Canada	2006	no	IFR
F6	IFR 06/005	Scallops	Canada	2006	no	IFR

*Originally from: CDC = C. Hatheway, Centers for Disease Control, USA; IFR = isolated at IFR; NCIMB = National Collection of Industrial, Food and Marine Bacteria, UK; NFPA = V. Scott, National Food Processors Association, USA; UH = M. Lindström, (University of Helsinki, Finland); UR= J. Crowther, Unilever Research, UK.E^NT^= no toxin gene detected.

The neurotoxin gene of each test strain was subtyped by sequencing diagnostic regions of each toxin type. A single region, B1, of the type B neurotoxin gene, predicted to be diagnostic for toxin subtype, was amplified by PCR and subjected to DNA sequencing. After end trimming, the sequence subjected to CLUSTALW analysis was 913 bp. The type B genes from the Group II strains studied here were all type B4 (Table 3). The B4 toxin type is generally associated with Group II *C. botulinum* strains and includes Eklund 17B (NRP). In clade 1, Eklund 17B (NRP) and its close relatives, the sequenced 913 bp diagnostic region of the type B toxin gene from Eklund 2B and IFR05/020 only differed from that of Eklund 17B by 2 bp and strain Colworth BL151 differed by 5 bp. The only two strains that had neurotoxin genes slightly diverged from others within the B4 group were Kapchunka B2 and Kapchunka B5, which were identical in sequence to each other but which differed from the neurotoxin gene of their closest relative, Eklund 17B (NRP), by 16 bp (1.75%). This does not constitute the basis for a new subtype but marks these two strains as divergent. The greatest difference observed between the type B toxin nucleotide sequences of any two Group I or Group II *C. botulinum* strains (Osaka 05 (putative B5) and Kapchunka B2 or Kapchunka B5), represented 61 of 913 bp, equivalent to 7%.

Two regions of the type E neurotoxin gene, predicted to be diagnostic for toxin subtype, were sequenced. After end trimming, the sequences for region ESTR1 were 641 bp, and those for ESTR2 were 626 bp. CLUSTALW (AlignX) analysis showed that each region distinguished some but not all of the subtypes; however combining both sets of data allowed an unambiguous interpretation. The type E strains were one of four toxin subtypes, E1, E2, E3 or E6 (Table 3). No examples were found for subtypes E4 and E5, which to date have only been described in strains of *C. butyricum*, nor for the more recently described subtypes E7 and E8 [12].

A single region of the type F neurotoxin gene was chosen for diagnostic sequencing. After end trimming this was 1,108 bp long. All five type F strains contained neurotoxin genes encoding subtype F6. PCR and subsequent DNA sequencing of the region spanning the *orfX2*/*p47* genes showed the two strains isolated from scallops in 2006 (IFR06/001 and IFR06/005) lacked the *botR* gene as has previously been shown for subtype F6 strain IBCA66-5463 [17]. PCR results suggest that *botR* is also missing from the toxin cluster of the three other type F strains isolated more than 30 years previously. All Group II *C. botulinum* strains contained a single neurotoxin gene, with no silent toxin genes identified.

### Clade specific CDS and physiology

From the microarray data, one hundred and forty CDSs were scored as present in Clade 1 strains but absent in Clade 2 and 3 strains using a Pearson correlation cut-off of 0.9. Of these, 135 did not have a predicted function and were therefore annotated as either hypothetical or putative proteins. The five CDSs with a strongly predicted function were ABC transporter ATP-binding protein (CB17B1783), aspartyl-tRNA synthetase (CB17B2424), two component response regulator (CB17B1780), GnaT-family acetyltransferase (CB17B2064) and phage protein (CB17B0899). Thirteen putative bacteriophage genes were additionally detected. Repeating the hierarchical clustering after removing CDSs specific to Clade 1 strains did not alter the way the clustering divided the strains into three Clades. This suggests that the separation of the Clade 1 strains was not solely dependent on the presence of common bacteriophage material in the strains in this clade.

The remaining type B strains were in Clade 2 along with all the type F strains. These strains had chromosomal DNA matching 80 to 87% of Eklund 17B (NRP) CDS probes. The type F strains were on a single branch but were as closely related to type B strains as some B strains were to each other. With the exception of the toxin cluster, there were no CDSs common to all type B strains that were absent from all type F strains.

All the type E strains and no type B or F strains were in Clade 3. Type E strains had chromosomal DNA matching 52 to 78% of Eklund 17B (NRP) CDS probes. A total of 577 CDSs present in all type B and type F strains were missing or diverged in all the type E strains using a Pearson correlation cut-off of 0.9.

Care must be taken when interpreting microarray results since an absence of a signal indicates only that the sequence used for the probe was either missing or diverged. A strain that appeared to lack a CDS on the microarray could have a similar sequence with nucleotide base changes in the probe area. Similarly, variation in CDS does not automatically mean there is variation at the protein level; as a result of codon redundancy the strains could be phenotypically similar without being genetically the same.

In order to predict physiological differences between strains in Clades 1 and 2 and those in Clade 3, CDSs specific to type B and type F strains were subjected to a TBLASTX search against the sequenced type E strain, *C. botulinum* Alaska E43. This procedure identified 154 CDSs that did not have an equivalent amino acid sequence in Alaska E43. Further analysis identified 33 amino acid sequences that were annotated for Group II *C. botulinum* strain Eklund 17B (NRP) but where a similar function had not been assigned to any CDS in the type E strains Alaska E43 or Beluga. These comprised several related to carbohydrate utilization including five chitinases, chitodextrinase, alpha amylase, beta-amylase, neopullulanase, maltose:maltodextrin transport system permease and starch binding protein. The TehB tellurite resistance protein was also identified. Tests for tellurite resistance and carbohydrate utilisation were carried out to determine if the microarray findings correlated with physiological differences between type B or F strains and type E strains.

**Figure 6:**
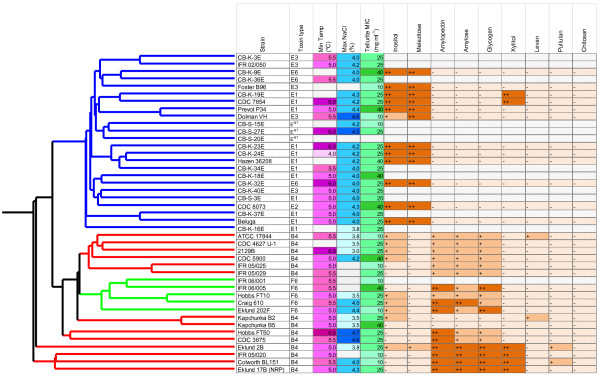
**Physiological characteristics of strains of Group II *C. botulinum.*** The minimum temperature and maximum NaCl concentration at which growth was observed, the minimum concentration of tellurite required to prevent growth and the ability to ferment selected carbohydrates was tested on strains representing different clades. Acid production was measured in a PY basal medium with 10 g l^-1^ added carbohydrate. A carbohydrate was considered to be fermented if the final pH was more than 0.5 units less than inoculated medium in the absence of carbohydrate. A pH reduction of 0.5-1.0 units was noted as acid production (+) and >1.0 was noted as strong acid production (++). For details of the hierarchical clustering dendrogram see Figure 5.

The minimum growth temperature and maximum NaCl concentration allowing growth in the test conditions, and the results of the tellurite MIC assay and the carbohydrate fermentation tests are shown in Figure 6. Although there is variability between the strains, differences in the minimum growth temperature, maximum NaCl concentration and tellurite resistance were relatively small compared to the range in bacteria as a whole. The range was 2.5°C in growth temperature and 1.7% in NaCl concentration. The mean MIC of tellurite was also similar for all strains, ranging from 10–40 mg ml^-1^. Bacteria are considered to be sensitive to tellurite if the MIC is 1-2 mg l^-1^ and to be resistant at 1000 mg l^-1^[18]. All the strains were of intermediate sensitivity. No relationship was observed between tellurite resistance and toxin type or Clade with the mean MIC of strains producing type B, E or F toxin being 25, 27 and 26 mg l^-1^ respectively. Similarly, there did not appear to be a clear relationship between toxin type and minimum growth temperature or maximum NaCl concentration for growth. In the conditions used in this test, all strains grew at 6.5°C and the lowest temperature at which growth was observed was 4.0°C. The median minimum growth temperature was 5.0°C for strains of each toxin type. On average the type E strains had a slightly higher tolerance to NaCl than type B and type F strains, with a mean maximum NaCl concentration of 4.1% and 3.9% respectively, but there was considerable overlap between the strains of each toxin type.

Different trends of carbohydrate utilisation were observed between strains in the three clades. All of the type E strains produced substantial quantities of acid from melezitose reducing the pH by more than 1 pH unit whereas no type F strains and only one type B strain, Eklund 2B produced acid and that strain only reduced the pH by 0.5 units. A similar but not identical response was observed for inositol fermentation with little or no acid production from type B or type F strains but large amounts of acid produced by all type E strains except Dolman VH. None of the type E toxin producing strains produced acid from amylopectin, amylose or glycogen whilst all the F strains and the majority of B strains did (Figure 6). Substantial acid was produced from xylitol by all type B strains in Clade 1 and two type E strains CB-K-19E and CDC 7854.

## Discussion

Previous studies have shown that Group II *C. botulinum* is a distinct species with strains forming a tight genetic group differing only in the type of neurotoxin formed: previous studies of 16S rRNA have shown they have 99.6-99.7% sequence similarity [19]. The results of the present study show that, although Group II *C. botulinum* types B, E and F strains are closely related, the genomic content is quite variable and the strains can be grouped into distinct subsets: The type B and type F strains appear to be closely related to each other with only toxin cluster CDSs being unique to either type while the type E strains formed a separate subset in the whole genome analysis. The dissimilarity is such that type B or F strains can be distinguished from type E strains on the basis of their whole genome alone, independently of their toxin cluster genes.

### Comparative genomic indexing of strains using Microarray

The microarray used in this study was composed of probes for 3524 CDS in Eklund 17B (NRP) with 1467 of these being detected in all the strains tested. The 58% of CDS that were missing or diverged in at least one test strain is greater than 37% reported for Group I *C. botulinum* strains [20] or 11-12% previously reported for a microarray analysis of Nordic Group I *C. botulinum* strains [21]. In the case of the Nordic strains part of this disparity is likely to relate to the strains being isolates from a narrow geographical area. Additionally some may be related to differences between the oligonucleotide based microarrays used in the present study and the PCR based microarrays used to study Group I *C. botulinum* strains. However, the low number of CDSs that could be detected as being shared by all strains tested illustrates there is considerable variability between strains of Group II *C. botulinum* at the nucleotide level. The present study supports the observation based on typing methods that genetic diversity between strains of Group II *C. botulinum* is greater than that between strains of Group I *C. botulinum*[2,22]

The clustering patterns obtained using the microarray data are consistent with other typing methods previously used to classify Group II *C. botulinum* strains [6,9,13]. An AFLP analysis by Hill et al. [6] also showed a very close relationship between Eklund 17B and Eklund 2B and separated Group II *C. botulinum* type B and type F strains from type E strains. Similar patterns were also observed in a dendrogram based on *Hin*dIII ribotyping [10] and AFLP banding patterns [7,23]. A study using flagellin diversity to characterise *C. botulinum* strains, which included 7 type B, 27 type E and 6 type F Group II strains, clustered strains into ten flaVA types based on the DNA sequence of a flagellin variable region [24]. They found that all the Group II type B and type F strains were flaVA group 9 while the E strains were of type 8 or 10. The agreement with other genetic based methods suggests genomic indexing using microarrays could be an effective method of typing Group II *C. botulinum*. The consistency with which type B and F strains are clustered separately from type E strains by a number of different typing methods demonstrates there is a marked difference between these two subsets which goes beyond the type of toxin cluster they contain. One important benefit of this genomic microarray study over previous studies based on banding patterns is that it provides information on the genetic nature of this difference.

Since the microarray was constructed using the sequence for *C. botulinum* Eklund 17B (NRP), it will have a greater ability to resolve the more closely related type B and F strains than the more distantly related type E strains. For type E strains, 2856 CDSs were present in at least one strain and 1556 CDSs were present in all type E strains, indicating that there were 1300 CDSs to which the response of type E strains was variable. The clustering of type E strains from the microarray data agrees with that observed in previous studies. For example, type E strains Beluga and CB-K-37E were closely related on the microarray dendrogram and clustered together in an analysis of type E strains by both MLST and AFLP [12]. This suggests that despite being based on the Eklund 17B genome, the microarray still contained a sufficient number of probes to variable CDSs to effectively type *C. botulinum* type E strains.

The strains tested were isolated from a variety of foods and environmental sources from a number of different locations over a wide chronological period. No relationship was evident between their original environment, geographical location or date of isolation and the clustering pattern. This lack of relationship has previously been reported for type E strains by MacDonald et al. 2011 [12] and is now shown also to be true for type B and type F strains. The clustering together of diverse strains isolated many years apart from distant locations suggests the genetic background of Group II *C. botulinum* is relatively stable.

The 1467 CDS detected in all strains using the microarray was substantially smaller than the conserved core geneset of 2521 genes that were identified between Eklund 17B (NRP), Beluga and Alaska E43 by reciprocal best match analysis of the genome sequences. Much of this difference may be explained by the fact that the microarray study compared 43 strains representing types B, E and F whereas the reciprocal best match analysis compared only three genomes including a single type B strain and no type F strain. This indicates the need for further genomic sequence information from these Group II *C. botulinum* strains.

### *C. botulinum* neurotoxin cluster genes and subtyping

Microarray analysis showed all the strains tested had a single neurotoxin encoding gene with the type B gene associated with *ha* cluster, and type E toxin genes and type F toxin genes associated with *orfX* cluster. All the strains of Group II *C. botulinum* scored positive for a single toxin gene type whereas strains of Group I *C. botulinum* have been shown to possess either one or two neurotoxin genes. The type B strains and the type F strains each had a single neurotoxin subtype.

It has been reported that the neurotoxin gene cluster has an evolutionary background distinct from a relatively stable genomic background for both Group I and Group II *C. botulinum* organisms [6]. There seems to be the potential for horizontal transfer of toxin genes between species with type B toxin genes being found in Group I *C. botulinum* and Group II *C. botulinum;* type E toxin genes in both Group II *C. botulinum* and *C. butyricum;* and type F toxin genes present in both Groups I and II *C. botulinum* and *C. baratii*. It has also been proposed that the similar gene arrangement of *orfx1*, *orfx2*, *orfx3* and *p47* in type A and type E toxin clusters suggests that gene transfer and recombination have occurred among type A and type E neurotoxin-producing clostridia [25]. It has also been observed that strains can lose the ability to produce toxin in laboratory culturing. It is then interesting to speculate why the present and previous studies appear to show a lack of transfer of toxin genes between Group II *C. botulinum* type E strains and the closely related Group II *C. botulinum* type B and type F strains, particularly when the toxin cluster is frequently on a mobile element and is seemingly easily lost. In this respect the toxin genes do not appear to be highly promiscuous and, to date, there is little evidence of horizontal gene transfer between Group II type B or type F and type E strains.

The potential for horizontal gene transfer in the genome of Eklund 17B (NRP) was examined using the program AlienHunter [26] to identify particularly high scoring regions in addition to visualisation of whole genome comparisons. The number of putative horizontally transferred genes was very low with only one region that presented a score indicating the possibility of being laterally transferred. This short region of higher GC content encodes a DNA helicase, a conserved hypothetical protein and a putative cell envelope protein comprising of two Pfam DUF11 domains of low score. This predicted protein shares some similarity with the C-terminal part of repetitive proteins from the aerobic sporeformer *Paenibacillus sp*.

Strains resembling Group II *C. botulinum* but that lack the neurotoxin gene have been isolated and, in the absence of specific nonemculture, are often called “type E like” strains [27]. The present study has revealed that some non-neurotoxigenic Group II *C. botulinum* strains are more like type B or F strains and than type E strains so it may be better to refer to all non-toxic strains as non-neurotoxigenic Group II *C. botulinum*.

### Clade specific CDSs and physiology

The genomic indexing study showed that type B and F strains fall into a separate subset from type E strains. Combining the microarray results with the genome sequence data of Group II *C. botulinum* Eklund 17B (NRP) and Alaska E43 allowed physiological differences between type B and F and type E strains to be predicted. However, the observed physiology did not necessarily match the predictions.

Analysis of the annotated genome for Eklund 17B (NRP) showed that all type B and type F strains tested carried *tehB,* a gene for tellurite resistance. However, this sequence was not present in any type E strain. It is a reasonable hypothesis that the presence of *tehB* would render type B and F strains more resistant to tellurite than type E strains. Physiological tests showed all strains were of intermediate tellurite resistance and that there was no direct relationship between toxin type and tellurite resistance. Therefore, the type E strains either have an alternative mechanism for tellurite resistance or they carry a version of *tehB* that is divergent from the Eklund 17B (NRP) *tehB* orthologue. Further analysis revealed two other genes that have been implicated in tellurite resistance, *terD*[28] and *cysK*[29] are present in Eklund 17B (NRP) and thus were included in the genomic indexing study. All the Group II *C. botulinum* strains tested contained *cysK*, the gene product of which is cysteine synthase which catalyses the last step in cysteine biosynthesis. The *terD* gene was identified in all of the four Clade 1 strains, one Clade 2 type B strains and two Clade 3 type E strains. The presence of these genes did not correlate with the observed tellurite resistance.

The data from the carbohydrate fermentation tests showed clear physiological differences between the type E toxin producing strains and the type B and type F toxin producing strains of Group II *C. botulinum*. These different patterns of carbohydrate fermentation could be helpful in identifying different subsets of Group II *C. botulinum*. Although differences in carbohydrate fermentation were reflected in the clades generated by the genomic indexing study, it was not possible to predict which specific carbohydrates would be used by analysis of the microarray results. Therefore, this study was unable to relate the genotype to the phenotype in terms of carbohydrate catabolism. The reason for the discrepancies may be partly due to shortcomings in the microarray data that shows only which CDSs are missing or divergent from that of the reference strain, and does not indicate any alternative or additional CDSs present in the test strain, and partly because the currently available annotation does not provide sufficient data for an accurate prediction of physiological behaviour. Physiological findings apparently disparate to the genetic data have been recently described in *Escherichia coli* O42 and may be as a result of a lack of general scientific data on a system and/or systems having the ability to compensate for the loss of genes by the use of others, or may result from a lack of transporters or ability of a specific substance to be taken up by individual strains [30].

Each of the type B and type F strains carried five CDSs annotated as chitinases whilst no such CDSs were identified by either microarray or BLAST in the type E strains. However, none of the type B, E or F strains fermented chitosan and Eklund 17B (NRP) failed to degrade chitin in an agar plate based activity assay (results not shown, for method see [31]). Further analysis is required to establish whether the sequences designated as chitinases are involved in degradation of chitin or other polysaccharides. Since chitin is composed of repeating units of N-acetylglucosamine (NAG), and NAG is present in the bacterial cell wall, the functions of these enzymes may involve degradation of bacterial cell wall components rather than of chitin itself. Similarly, analysis of the pathways in the KEGG database for the two fully sequenced strains of Group II *C. botulinum,* Eklund 17B (JGI) and Alaska E43, did not necessarily correspond to the observed physiological data. For example, there was not an obvious explanation for the difference in inositol or xylitol utilisation between the type B strain Eklund 17B (JGI) and the type E strain Alaska E43 as the enzymes required in the pathway for their utilisation were not found in either. However, *C. botulinum* Eklund 17B (JGI) did contain an open reading frame annotated as glucose-1-phosphate cytidylyltransferase, an enzyme used in the conversion of starch and glycogen to CDP-glucose, which is used in amino sugar and nucleotide sugar metabolism. The genome of Alaska E43 did not contain a CDS annotated as being for for this enzyme and the type E strains were unable to ferment amylose, amylopectin or glycogen.

Failure to find any relationship between annotation and the physiological differences observed between the different cluster types highlights that, as a predictor of the underlying physiology and metabolism, automated annotation shows clear discrepancies. These point to the need for better curation of annotations in the future, in the light of experimental data. Current annotations are subject to transitive catastrophe, where a mistaken annotation can easily become propagated through many future annotations. Taken together with lack of scientific knowledge on particular systems, of regulatory and transport processes, it is clear that in some cases the currently available annotation in conjunction with KEGG mapping data shows particular discrepancies with respect to the metabolic pathways predicted in the organism and the actual metabolism seen in culture.

The observation that there are genetic and physiological differences between type B and F strains and type E strains could be important to the risk of food poisoning. The ability of type B and F strains but not type E strains to ferment amylose, amylopectin or glycogen is important for microbiological food safety as starch is a major component of many foods. Similarly, information on strain variability in minimum growth temperature and the maximum NaCl concentration or water activity allowing growth is important for improved quantitative risk assessment. The average temperature of chilled food at retail in the UK is 4 – 6°C and the average temperature in domestic refrigerators is 6.6°C [4]. Information on the number, or type, of strains able to grow at these temperatures is necessary to be able to predict the potential for growth in chilled foods and thereby control product safety. Previous workers have reported better growth at low temperatures of type F strains than other Group II strains [32,33] although a recent study reported that type F strains of Group II *C. botulinum* have a higher minimum growth temperature than type B or type E strains [23]. The present study did not identify an association of toxin type with stronger growth at chill temperature. Considering the increasing sales of chilled food [4] and the importance of chill temperature for food safety, further analysis seems justified.

## Conclusions

A genomic microarray was created and used to characterise strains of Group II *C. botulinum*. This method was shown to be capable of typing strains of Group II *C. botulinum*. The results obtained from the genomic indexing were compared with physiological tests for minimum growth temperature, maximum NaCl concentration for growth, carbohydrate utilisation and sensitivity to tellurite. Although Group II *C. botulinum* strains form a tight genetic group, genomic and physiological analysis indicates the strains fall into two subsets: all type B strains and type F strains are in one subset and all type E strains in the other. No evidence of recent transfer of toxin genes between these two groups was found.

No strong relationship between toxin type and minimum growth temperature or maximum NaCl concentration for growth was observed. However, data from the carbohydrate fermentation tests showed different patterns of carbohydrate fermentation between the type E toxin producing strains and the type B and F toxin producing strains of Group II *C. botulinum* which could be helpful in identifying these different types. Coding sequences that were present in all type B and F strains but not in type E strains were identified but the current level of annotation for this organism (and a lack of experimental evidence addressing metabolism) meant it was not possible to translate any of these differences directly into observed physiological characteristics. The differences observed between the type E strains and the type B and F strains indicate that there is more genetic and physiological variability in Group II *C. botulinum* strains than was previously thought.

## Methods

### Bacterial strains and growth

Forty-three strains of Group II (non-proteolytic) *C. botulinum* were used in this study, 14 type B, 24 type E and 5 type F. These strains, chosen to represent a range of the available isolation environments and geographical locations and isolated over a period of 72 years, are shown in Table 3. Before use, the purity of all strains was confirmed by growth on PYGS and reinforced clostridial Medium (RCM) with 5% (w/v) skim milk agar plates incubated under aerobic and anaerobic atmospheres.

### Sequencing of Eklund 17B (NRP) genome

*C. botulinum* Eklund 17B (NRP) was initially sequenced using 454 technology (SE FLX run). This run produced 101,205,149 bp in 919,403 reads giving an average read length of 110 bp. The reads were assembled using Newbler 01.51.02 to give 186 contigs, covering a total of 3,762,410 bp. The average contig size was 20,228 bp; the largest contig was 129,200 bp. Overall coverage was 26 times. Four genomic shotgun libraries were also sequenced using dye terminator chemistry on ABI 3700 automated sequencers. Combined assembly of capillary and 454 sequence produced contigs which gave an average genome size of 3,852,568 bp. Eklund 17B (NRP) DNA was also analysed by Illumina SE 37 cycle sequencing, yielding 151,042,368 bp (representing 38× coverage). An Eklund 17B genome sequence from the Joint Genome Institute, USA was later published (Eklund 17B (JGI)). Our sequence was subsequently designated Eklund 17B (NRP) to allow distinction between these two genome sequences.

Gene prediction was carried out using Glimmer3 and manually curated. Annotation was initially carried out using an annotation transfer procedure from a database of previously manually curated annotations. This resulted in annotation coverage of approximately 85% of the geneset. The remaining 15% of the CDSs were annotated automatically using RAST with some additional manual curation. The complete finished sequence and annotations for the chromosome and plasmid were submitted to EMBL and are available as FR745875 and FR745876, respectively.

### Microarray construction

The microarray was designed by Oxford Gene Technology (OGT, Oxford, UK) and slides were printed by Agilent Technologies. The microarray was designed from the Eklund 17B (NRP) initial 454 sequence data before the sequence finishing was complete. All CDSs greater than 800 bp were extracted and used to build a strain-specific model based on this CDS set. For Glimmer processing of the Eklund 17B (NRP) 454 sequences, all contigs were joined together to form one supercontig, with start and stop codons added in all reading frames as spacers in between the original contigs. Four reference genomes were used for microarray CDS annotation and in order to bench mark Glimmer performance: *Clostridium acetobutylicum* ATCC 824 (AE001437.1); *Clostridium perfringens* strain 13 (BA000016.3); *Clostridium tetani* E88 (AE015927.1); *Clostridium botulinum* ATCC 3502 (AM412317). The Glimmer predictions for this supercontig were mapped back onto the original 454 contigs to avoid designing probes overlapping with parts of the inserted artificial sequences. This approach yielded 3974 CDSs. Additionally, a BLAST comparison of the 12599 CDSs from all four reference genomes was carried out against all 454 contigs to identify all possible orthologues. After filtering out regions not covered by Glimmer predictions, 140 sequences more than 60 bp in length were identified. The 4114 sequences detected by Glimmer and BLAST were used to design up to three 60 nucleotide probes for each CDS. A total of 10,817 probes together with 10,817 mismatch controls were printed on microarray slides in randomised order with a border of positive control probes. This initial microarray was used to experimentally validate the probe set using Eklund 17B (NRP) genomic DNA and enable selection of an optimised probe set.

The optimised probe set comprised 4160 probes to unique sites in CDS and RNA features of the chromosome and plasmid of Group II *C. botulinum* Eklund 17B (NRP). A second microarray for *C. botulinum* neurotoxin cluster genes was also constructed. This consisted of 94 probes representing CDSs of *C. botulinum* neurotoxins (Types A, C, D, E and F) and associated proteins (i.e. p47, orfX1, orfX2, orfX3, botR) that had been reported for other sequenced strains of *C. botulinum*. This array was used detect toxin cluster CDSs but the results were not used in the comparative genomic indexing analysis.

### Probe preparation and hybridisation

Genomic DNA was purified from exponentially growing cells as previously described [31] and labelled with fluorophore using the BioPrime® DNA Labelling System (Invitrogen). The DNA was digested with Dra1 (Promega), analyzed by standard gel electrophoresis in 1.5% agarose, cleaned using a BioPrime® CGH purification module, and labelled with Cy5-dUTP or Cy3-dUTP fluorescent nucleotides (GE Healthcare, UK). Unincorporated nucleotides were removed using a DyeEx 2.0 spin column (Qiagen) before the levels of DNA and label were measured and base:dye ratio was calculated using the program for CyDyes on a NanoDrop spectrophotometer (Thermo Scientific).

Each strain was hybridized on at least two microarrays. For each hybridization reaction, Cy3-labelled genomic DNA from the reference Eklund 17B (NRP) strain was mixed with Cy5-labelled genomic DNA from the test strain. Reference and test DNA (1 μg of each) were mixed in a total of 250 μl hybridisation solution (containing 25 μl 10× Agilent blocking agent, 125 μl Agilent hybridisation buffer and water to volume), heated at 100°C for 3 min followed by 37°C for 30 min then added to a microarray slide. Hybridisation was performed at 60°C for at least 18 h. Hybridised slides were washed by rotation for 5 min in 6×SSPE, 0.005% lauryl sarcosine, followed by 1 minute in 0.06% SSPE, 0.18% PEG200, 1 min in acetonitrile and 30 s in stabilisation and drying solution (Agilent).

Processed slides were scanned using a GenePix 4000B laser scanner (Axon Instruments, CA, USA) at 532 nm (Cy3) and 635 nm (Cy5) excitation wavelengths with a 10 μm resolution. The images obtained were initially processed using the GenePix Pro v.6.0 software supplied with the scanner. Features were identified and any that were poor were excluded from later analysis.

### Microarray Data analysis

Data analysis was performed using GeneSpring version 7 (Agilent Technologies). Data were processed by dividing signal values for the test sample by those of the control channel and then normalized to the 660 probes whose values were within 8-fold of that for the Eklund 17B (NRP) control channel for all the strains tested. CDS were considered as present if intensity of the spot was greater than that of the background plus 3SD and the intensity ratio of test to control sample was greater than 0.125. Genomic relationships were assessed by hierarchical clustering using the Pearson correlation clustering parameter with average linkage on averaged replicate samples. To find CDSs related to specific clades, an artificial “expression profile” was created where the strains in the clades of interest had an expression level of 1 and those in other clades an expression level of 0. GeneSpring was then used to find CDSs with similar profiles with a Pearson correlation of 0.9 or more. Annotated CDSs that were found to be specific to Clade 1 and 2 strains in the GeneSpring analysis were tested for the presence of equivalent proteins in type E strains by performing a TBLASTX search of the Group II *C. botulinum* Alaska E43 and Beluga genome sequences in the NCBI database.

### Physiological tests

#### Tellurite resistance of Group II Clostridium botulinum strains

The minimum inhibitory concentration (MIC) of tellurite was determined for each of the 43 Group II *C. botulinum* strains used in the microarray comparative genomics study. Each strain was grown for 16 h in PYGS medium at 30°C, and then diluted to an OD600nm of 0.2 in anaerobic buffered peptone water. Aliquots (10μl) of diluted culture were spotted onto PYGS agar plates containing potassium tellurite (K_2_TeO_3_, Fluka) at concentrations of 100, 85, 70, 55, 40, 25, 10, 5 and 0 mg l^-1^ and incubated at 30°C for 72 h in an anaerobic cabinet under a headspace of 5% CO_2_: 10% H_2_: 85% N_2_. The lowest concentration of tellurite that completely inhibited growth was defined as the MIC for that strain. MIC assays were performed in triplicate.

#### Carbohydrate fermentation

Thirty-three strains of Group II *C. botulinum*, chosen to represent different toxin types and the three different clades determined from the microarray analysis, were tested for their ability to ferment a range of carbohydrates. The carbohydrates tested were myo-inositol (Fluka 57570), D-(+)-melezitose monohydrate (Fluka 63620), amylopectin from maize (Sigma 10120), amylose from potato (Fluka A0512), glycogen from bovine liver (Type IX, Fluka G0885), xylitol (Fluka X3375), levan from *Erwinia herbicola* (Sigma L8647), pullulan from *Aureobasidium pullulans* (Sigma P4516) and chitosan (Aldrich 448877).

The basal PY medium consisted of (l^-1^): Peptone, 10 g; yeast extract, 5.0 g; sodium chloride, 5.0 g; cysteine HCl, 0.5 g; hemin solution (0.5 mg ml^-1^) 10.0 ml; vitamin K1 solution (1μl ml^-1^) 0.2 ml; resazurin solution (0.2 mg ml^-1^) 5.0 ml. Carbohydrate was added at 10 g l^-1^ as required. Broths were inoculated with 10 μl of turbid, exponential phase culture and incubated at 30°C for 48 h. After incubation, the pH was measured. A carbohydrate was considered to be fermented if the reduction in pH was 0.5 units greater than that observed in inoculated medium in the absence of carbohydrate. A pH reduction of 0.5-1.0 units was considered weak acid production and >1.0 strong acid production.

#### Ability of strains to grow at low temperature or tolerate NaCl

The ability of Group II *C. botulinum* strains to grow at refrigeration temperatures or in media containing different NaCl concentrations was tested using vials of PYGS medium inoculated with spores of appropriate strains. Spores were prepared by growing the cultures in Robertson’s cooked meat medium (CMM) for 7 days at 20°C. The spores were harvested using 10 μm Vectaspin 20 centrifuge filters (Whatman), washed five times by centrifugation in cold 0.85% saline (2000 g, 20 min, 4°C), resuspended in 0.5 ml saline, enumerated on PYGS agar and refrigerated until required. Spores were activated at 60°C for 20 min immediately before use and 10 μl spore suspension containing 10^6^ spores were added to each triplicate tube containing 20 ml PYGS broth.

The temperature study used 20 ml PYGS broth, cooled to 2°C and inoculated with 10^6^ spores of the appropriate test strain. Inoculated tubes were incubated at 3.0, 4.0, 5.0, 5.5, 6.0, 6.5, 7.0, 8.0, 9.0 and 10.0°C. For the NaCl study, PYGS was prepared containing 0, 3.0, 3.5, 3.8, 4.0, 4.2, 4.3, 4.4, 4.5, 4.6, 4.8 or 5.0% NaCl w/v. Vials were incubated at 30°C for up to 90 days and observed weekly for signs of turbidity or gas formation. Growth/no-growth was also confirmed by measuring toxin production using a ELISA for the appropriate neurotoxin as vials became turbid, or at the end of the incubation period.

### Toxin subtyping

Comparison of neurotoxin gene sequences representing different subtypes was used to design PCR primers for amplification of diagnostic regions. GenBank Accession Numbers for Type B subtypes were: (subtype B1) EF028399, EF028396; (subtype B2) AB302854, EF033128, EF051571; (subtype B3) EF028400; (subtype B4) EF051570; (subtype B5) EF028397; (subtype B6) AB302852; (subtype B7) JQ354985. Subtype classifications for B6 and B7 are as used by Kalb et al. [34]. Accession Numbers for Type E subtypes were: (subtype E1) X62683, X62089; (subtype E2) EF028404; (subtype E3) AM695760, U70780; (subtype E4) AB088207, AB039264; (subtype E5) AB037714, AB037704; (subtype E6) AM695752, AM695765; (subtype E7) JN695729; (subtype E8) JN695730. Accession numbers of Type F strains were: (subtype F1) GU213203; (subtype F2) Y13631; (subtype F3) GU213218; (subtype F4) GU213210, GU213224; (subtype F5) GU213217, GU213225; (subtype F6) GU213228, GU213230; (subtype F7) GU21323, GU213233.

Primers for amplification of a single region of approximately 1 kb diagnostic region of Type B neurotoxin genes were; BF1 GGCTGGAAAATATCTATTAGGGGT and BR1 TTATTCAATCCATCCTTCATCTTT (primer binding coordinates for the type B1 neurotoxin gene of strain Okra are 2881–2904 and 3853–3876, respectively). PCR was performed under the following conditions using 50 ng genomic DNA as template; each 50 μl reaction contained 25 μl HotStarTaq Master Mix (Qiagen), 1 μl 50 mM Mg Cl_2,_ and 2.5 μl each primer (10 μM). Cycling parameters were: 95°C, 15 min; 35 cycles each of 94°C, 20 s; 57°C, 30 s; 72°C, 2 min, followed by one cycle each of 72°C, 5 min, 10°C, 16 h. PCR products were checked by agarose gel electrophoresis, purified and sequenced. DNA sequences were aligned using the Vector NTI Advance 11.0 AlignX programme and compared with those of the reference subtypes listed above. Primers were designed for amplification of two 700 bp diagnostic regions of Type E neurotoxin genes: region ESTR1; EF1 GCAATCACGGTTTTGGATCAATAG, ER1 GATTTTTTTTACTAGTCCTCTACCTG and region ESTR2; EF2 GATGGAAAGTATCTCTTAATCATAATG, ER2 GATTAAATCTATTGCCAGATGATG (primer binding coordinates for the type E3 neurotoxin gene of strain Alaska are (region ESTR1) 530–553 and 1208–1233; (region ESTR2) 2855–2881 and 3542–3565, respectively). Type F strains were subtyped using a single diagnostic 1.1 kb region of the Type F neurotoxin gene. The PCR primers were: FF4: GTAATAAGAGATTTTACCACTG; and FR7: TCAATTTCTGTTTTATCTAATTCCG (primer binding coordinates for the type F1 neurotoxin gene of strain Langeland are 1792–1813 and 3209–3233, respectively). In addition PCR primers were designed to allow sequencing of the *orfX2* – *p47* region. These primers were: ORFX2R1: TTATGAAATTTGTAATAAATTTTCTTCAT; P47R3: CCATTAATAATTTCCCAATTATCAA; P47R4: GAATATATACGTTCATATCCTGTAAG (designating the last nt of the ORF-X3 gene stop codon as nt position 1, primer binding coordinates for the type F1 neurotoxin gene cluster of strain Langeland are 3777-3805, 6421-6445 and 7194-7219, respectively).

## Competing interests

The authors declare that they have no competing interests.

## Authors’ contributions

MWP obtained funds for, initiated and conceived this study. SCS and MDW carried out the genomic indexing work. ATC liaised with OGT on microarray design. SCS carried out the microarray analysis with a contribution from ATC. ATC carried out toxin subtyping and some of the genome sequence comparisons. MS co-ordinated sequencing of the genome and MS and LC annotated the genome. LC submitted sequence data to EMBL and carried out the PHAST, Alien hunter and REPuter analysis and the majority of the genome sequence comparisons. SCS initiated and coordinated the physiological tests. SCS and MDW performed the low temperature and NaCl growth study. MDW carried out the tellurite study and EW the sugar utilisation tests. SCS coordinated the writing of the manuscript with all authors providing critical feedback. All authors read and approved the final manuscript.

## Supplementary Material

Additional file 1 SNPs, indel and ribosomal RNA assembly differences between the Eklund17B (NRP) and Eklund 17B (JGI) sequences. Click here for file
